# A Preliminary, Multicenter, Prospective and Real World Study on the Hemostasis, Coagulation, and Safety of Hemocoagulase Bothrops Atrox in Patients Undergoing Transurethral Bipolar Plasmakinetic Prostatectomy

**DOI:** 10.3389/fphar.2019.01426

**Published:** 2019-11-27

**Authors:** Bing-Hui Li, Zhao-Jun Yu, Chao-Yang Wang, Hao Zi, Xiao-Dong Li, Xing-Huan Wang, Xuan-Yi Ren, Tong-Zu Liu, Hang Zheng

**Affiliations:** ^1^Department of Urology, Zhongnan Hospital of Wuhan University, Wuhan, China; ^2^Medical Department of Graduate School, Nanchang University, Nanchang, China; ^3^Center for Evidence-Based Medicine, Institute of Evidence-Based Medicine and Knowledge Translation, Henan University, Kaifeng, China; ^4^Department of Urology, Huaihe Hospital of Henan University, Kaifeng, China; ^5^Department of Urology, Kaifeng Central Hospital, Kaifeng, China

**Keywords:** benign prostatic hyperplasia, transurethral bipolar plasmakinetic prostatectomy, hemocoagulase bothrops atrox, hemostasis, blood transfusion, coagulation, real world study

## Abstract

**Objective:** To evaluate the hemostasis and coagulation effect of Hemocoagulase Bothrops Atrox in benign prostatic hyperplasia (BPH) patients undergoing transurethral bipolar plasmakinetic prostatectomy (TUPKP).

**Methods:** This study adopted a multicenter, prospective, and real world design. BPH patients undergoing TUPKP were divided into two groups according to whether they adopted Hemocoagulase Bothrops Atrox (group B) or not (group A) during perioperative period. The electronic clinical data on every included subject, including the international prostate symptom score (IPSS) and the quality of life scale (QoL), maximum urinary flow rate (Qmax), complete blood count, coagulation screening test and adverse events, were measured and compared between the two groups.

**Results:** Finally, 695 patients, 443 in group A and 252 in group B were included. Baseline characteristics showed no significant difference between two groups. In group A, compared with baseline, IPSS decreased 15.66 (95% CI = −16.45 to −14.87), QoL decreased 3.08 (95% CI = −3.30 to −2.87), prothrombin time prolonged 1.02 s (95% CI = 0.56 to 1.48), while white blood cells, neutrophils, lymphocytes, and hemoglobin also significantly changed; white blood cells, neutrophils and platelets increased, while lymphocytes decreased by 0.14×109/L (95% CI = −0.21 to −0.08) before discharge. In group B, compared with baseline, IPSS decreased 16.12 (95% CI = −17.02 to −15.21), QoL decreased 3.32 (95% CI = −3.56 to −3.07), and white blood cells, neutrophils, lymphocytes, and hemoglobin were also significantly changed, along with white blood cells and lymphocytes that tested before discharge (p < 0.01); however, prothrombin time was not significant prolonged (MD= 0.48, 95% CI = −0.05 to 1.01). When compared with group A and group B, the average hospitalization time in group A was longer than group B (p < 0.01), transfusion risk was similar in the two groups (OR = 1.582, 95% CI = 0.552 to 4.538). Parameters had no substantial difference between the two subgroups whether prostate volume was more than 80 mL or not.

**Conclusion:** Our study indicated that Hemocoagulase Bothrops Atrox can shorten the prothrombin time, hospitalization time and is probably safe among BPH patients undergoing TUPKP, exhibiting fine hemostasis and coagulation efficacy, and would not be influenced by prostate volume.

## Introduction

Benign prostatic hyperplasia (BPH) is a leading cause of lower urinary tract symptoms (LUTS) and a common benign disease among males, showing an upward prevalence with age. There are multiple options in treating BPH, including drug, surgical and minimally invasive options (Vuichoud and Loughlin, 2015; Chughtai et al., 2016). Transurethral resection of the prostate (TURP) is a common method in treating BPH, but perioperative complications, such as intraoperative bleeding and transurethral resection syndrome (TURS), still exhibit a relatively high incidence. In recent years, transurethral bipolar plasmakinetic prostatectomy (TUPKP) has been used in this field, reaching better hemostasis and showing less TURS than TURP (Ran et al., 2013). However, prostate-surrounding tissue contains large venous sinuses, which tend to breach during surgery, thus leading to complications such as bleeding and affecting the operation (de Medicis, 2014). Hemostatic agent can be used during perioperative period to reduce bleeding. Reportedly, tranexamic acid is beneficial for reducing perioperative blood loss in TURP (Meng et al., 2019). But rarely studies clarified the role of thrombin in TUPKP. Hence, this study aimed to evaluate the effect and safety of a commonly used hemostatic agent (Hemocoagulase Bothrops Atrox for injection) (Wei et al., 2010; Lu et al., 2017) in BPH patients with TUPKP, through examining prostate indicators and hematological parameters.

## Methods

### Study Design and Subjects

This study adopted a multicenter, prospective, and real world design for preliminary investigation. The subjects were selected from the Bladder Cancer and Benign Prostatic Hyperplasia Study in Chinese Population (BPSC), a prospective study reported previously (Zeng et al., 2018a; Zeng et al., 2018b; Zeng et al., 2018c; Wu et al., 2019; Zhao et al., 2019; Zi et al., 2019), which from September 2016 to November 2018. Eligible patients were confirmed with BPH and underwent TUPKP. Patients with urinary malignancies, urinary tract infections and abnormal coagulation function were excluded. Eventually included patients were divided into two groups according to whether they used Hemocoagulase Bothrops Atrox for Inject (brand name: baquting; production lot number: H20041419) (group B) or not during perioperative period (group A). Electronic clinical data on every included subject were retrospectively analyzed. This study was reviewed and approved by the Committee for Ethical Affairs of the Zhongnan Hospital of Wuhan University (Approval No. 2016028). All participants signed written informed consent before enrollment.

### Measurements and Data

Detailed medical histories for all patients were collected, including age, height, weight, body mass index (BMI), history of smoking, and history of alcohol intake. Physical examination results and detailed medical data were recorded, including clinical blood pressure, fasting blood glucose (FBG, ng/mL), prostate ultrasound, maximum urinary flow rate (Qmax), complete blood count (CBC), and coagulation screening test results. In addition, international prostate symptom score (IPSS) and the quality of life (QoL) scale were used to assess LUTS and perceptions of quality of life.

BMI (kg/m2) was calculated through weight in kilograms divided by height in meters squared; systolic blood pressure (SBP, mmHg) and diastolic blood pressure (DBP, mmHg) were measured following the way recommended by the American Heart Association guidelines (Pickering et al., 2005). The largest anteroposterior height (H, cm), transverse width (W, cm), and cephalocaudal length (L, cm) of prostate were measured through prostate ultrasound, and prostate volume (PV, mL) was calculated using the ellipsoid formula $PV=\frac{\pi }{6}\times H\times W\times L$ (Eri et al., 2002). Qmax (ml/s) was measured via flow rate test, along with IPSS and QoL, and recorded as indicators for assessing the patient’s prostate symptom. White blood cells (WBC, ×109/L), neutrophils (×109/L), platelets (×109/L), lymphocytes (×109/L) and hemoglobin were determined based on CBC. The results of activated partial thromboplastin time (APTT, s), prothrombin time (PT, s), thrombin time (TT, s) and fibrinogen (g/L) level were obtained from routine blood coagulation tests. The results of IPSS, Qmax, QoL, APTT, platelets, TT and fibrinogen were recorded at the admission and after operation, while those for WBC, neutrophils, platelets, lymphocytes and hemoglobin were recorded at the admission, after operation and before discharge. The data recorded at the admission were considered as baselines. In addition, hospitalization time was also extracted.

### Statistical Analysis

Continuous variables were expressed as mean and standard deviation (SD), while categorical ones as percentage. We compared age, height, weight, BMI, PV, history of smoking, history of alcohol intake, SBP, DBP, FBG, and hospitalization time between group A and group B. Student’s t-test was used for continuous variables and chi-squared tests for categorical ones. Adjusted mean changes in IPSS, Qmax, QoL, APTT, PT, TT, and fibrinogen level after operation from baseline, along with 95% confidence interval (95% CI), were calculated. WBC, neutrophils, platelets, lymphocytes and hemoglobin levels after operation and at discharge were compared with baseline ones, and mean changes were calculated. Meanwhile, two subgroups were divided with PV at 80 ml as the threshold, and the mean difference in each parameter was calculated separately. Differences in mean change between group A and group B were calculated. In addition, the association between blood transfusion and hemostatic agent during the operation was explored. We computed odds ratios (OR) and 95% CI using binary logistic regression model or ordinal logistic regression model. All effect values except subgroup were adjusted for age, BMI, PV, history of smoking, history of drinking, SBP, DBP and FBG. We only offered unadjusted subgroup results here without further analysis. All analyses were carried out using the SAS software, version 9.4 TS1M6 (SAS Institute Inc, Cary, NC).

## Results

### Participants’ Characteristics

A total of 695 patients were included in this study. Of the cases, 443 did not use Hemocoagulase Bothrops Atrox for Inject (group A) while 252 did during the perioperative period (group B). The baseline characteristics of all patients were presented in **Table 1**. The mean SBP was 134.28 ± 17.14 mmHg and 131.53 ± 16.00 mmHg (p = 0.04) for the two groups, respectively. The mean age reached 72.76 ± 7.60 years and 71.94 ± 7.24 years for the two groups, without statistically significant difference (p = 0.17). Likewise, there was no significant difference between the two groups in height, weight, BMI, PV, history of smoking, history of alcohol intake, DBP or FBG (all p > 0.05).

**Table 1:** Baseline characteristics of benign prostatic hyperplasia patients not use and use Hemocoagulase Bothrops Atrox for inject.

Baseline	Without use (Group A) (n=443)	With use (Group B) (n=252)	*P-value*
Age (years)	72.76 ± 7.60	71.94 ± 7.24	0.1665
Prostate volume (mL)	68.19 ± 37.18	62.75 ± 37.57	0.0652
Prostate volume (mL)			0.4107
>80 mL	138(31.15%)	181(71.83%)	
<=80 mL	305(68.85%)	71(28.17%)	
Height (cm)	168.46 ± 5.82	168.63 ± 5.33	0.7070
Weight (kg)	65.95 ± 10.60	66.47 ± 11.52	0.5569
Body mass index (kg/m^2^)	23.26 ± 3.43	23.35 ± 3.95	0.7625
History of smoking (n[%])	122(35.57%)	76(30.16%)	0.166
History of alcohol intake (n[%])	84(24.56%)	67(26.91%)	0.518
Systolic blood pressure (mmHg)	134.28 ± 17.14	131.53 ± 16.00	0.0381
Diastolic blood pressure (mmHg)	79.39 ± 10.36	78.57 ± 10.57	0.3231
Hypertension status (n[%])			0.147
No	373(85.35%)	224(89.24%)	
Yes	64(14.65%)	27(10.76%)	
Fasting blood glucose (ng/mL)	5.36 ± 1.51	5.41 ± 1.32	0.6929

### Overall Results

**Table 2** showed prostate indicators, CBC and coagulation screening test results for the groups A and B. The results of prostate indicators, CBC and coagulation screening test were also compared with baseline ones.

**Table 2:** Effects of Hemocoagulase Bothrops Atrox for inject on prostatic parameters and biochemical indexes in two groups.

Outcomes	Group A			Group B			Adjusted difference in mean change (95% CI)	***p***
	Baseline	Mean change (95% CI)	*P*	Baseline	Mean change (95% CI)	*p*		
**After operation**								
IPSS	23.25 ± 6.56	−15.66(−16.45, −14.87)	<0.001	24.39 ± 5.88	−16.12(−17.02, −15.21)	<0.001	0.45(−0.54,1.44)	0.368
Qmax (ml/s)	7.59 ± 5.71	4.26(−2.32,10.85)	0.2	9.06 ± 5.32	5.85(−1.47,13.16)	0.115	−1.58(−8.32,5.16)	0.641
Quality of Life	4.93 ± 0.86	−3.08(−3.30, −2.87)	<0.001	4.75 ± 1.00	−3.32(−3.56, −3.07)	<0.001	0.23(−0.03,0.50)	0.085
White blood cells (×10^9^/L)	6.36 ± 1.98	3.09(2.63,3.56)	<0.001	6.43 ± 2.35	2.99(2.47,3.50)	<0.001	0.11(−0.44,0.65)	0.699
Neutrophils (×10^9^/L)	4.22 ± 1.85	3.45(3.00,3.91)	<0.001	4.23 ± 2.24	3.40(2.91,3.90)	<0.001	0.05(−0.47,0.57)	0.854
Platelets (×10^9^/L)	190.01 ± 59.99	−5.43(−11.70,0.85)	0.09	180.03 ± 60.81	−4.08(−10.95,2.78)	0.243	−1.34(−8.65,5.96)	0.718
Lymphocytes (×10^9^/L)	1.56 ± 0.95	−0.35(−0.45, −0.25)	<0.001	1.48 ± 0.53	−0.42(−0.54, −0.31)	<0.001	0.07(−0.05,0.19)	0.224
Hemoglobin (g/L)	134.73 ± 17.20	−10.36(−12.28, −8.45)	<0.001	131.24 ± 15.56	−11.57(−13.67, −9.47)	<0.001	1.21(−1.02,3.44)	0.288
APTT (s)	33.24 ± 5.08	0.67(−1.60,2.95)	0.549	32.41 ± 4.60	0.99(−1.63,3.62)	0.445	−0.32(−3.41,2.77)	0.834
Prothrombin time (s)	12.62 ± 6.06	1.02(0.56,1.48)	<0.001	11.56 ± 1.48	0.48(−0.05,1.01)	0.074	0.54(−0.09,1.17)	0.089
Thrombin time (s)	15.73 ± 2.05	−0.55(−2.43,1.32)	0.547	14.68 ± 2.34	−0.10(−2.38,2.18)	0.928	−0.45(−2.82,1.91)	0.694
Fibrinogen (g/L)	3.24 ± 0.84	0.06(−0.32,0.44)	0.75	3.29 ± 0.91	−0.22(−0.68,0.23)	0.319	0.28(−0.22,0.79)	0.262
**Before discharge**								
White blood cells (×10^9^/L)	6.36 ± 1.98	0.35(0.00,0.69)	0.047	6.43 ± 2.35	0.17(−0.18,0.52)	0.333	0.17(−0.20,0.55)	0.365
Neutrophils (×10^9^/L)	4.22 ± 1.85	0.39(0.08,0.70)	0.013	4.23 ± 2.24	0.18(−0.14,0.49)	0.269	0.21(−0.13,0.55)	0.221
Platelets (×10^9^/L)	190.01 ± 59.99	23.43(13.77,33.10)	<0.001	180.03 ± 60.81	14.50(4.58,24.42)	0.004	8.93(−1.71,19.57)	0.1
Lymphocytes (×10^9^/L)	1.56 ± 0.95	−0.14(−0.21, −0.08)	<0.001	1.48 ± 0.53	−0.15(−0.22, −0.08)	<0.001	0.01(−0.07,0.08)	0.888
Hospitalization time (days)	8.20 ± 3.09			6.56 ± 3.60				<0.001

CI, confidence interval; IPSS: International Prostate Symptom Score; Qmax, maximum urinary flow rate; APTT, activated partial thromboplastin time.

Accordingly, IPSS decreased 15.66 (95% CI = −16.45 to −14.87, p < 0.01), QoL decreased 3.08 (95% CI = −3.30 to −2.87, p < 0.01), PT prolonged 1.02 s (95% CI = 0.56 to 1.48, p < 0.01), while WBC, neutrophils, lymphocytes, and hemoglobin also changed significantly in group A (p < 0.001). However, Qmax, platelets, APTT, TT and fibrinogen were not significantly different from baseline values (p > 0.05). CBC was re-examined before discharge. The results showed that compared with baseline figures, WBC, neutrophils and platelets increased while lymphocytes decreased 0.14×109/L (95% CI = −0.21 to −0.08, p < 0.01), all statistically significant (p < 0.05).

In group B, IPSS decreased 16.12 (95% CI = −17.02 to −15.21, p < 0.01), QoL decreased 3.32 (95% CI = −3.56 to −3.07, p < 0.01), while WBC, neutrophils, lymphocytes, and hemoglobin were also changed significantly, along with WBC and lymphocytes that tested before discharge (p < 0.01). However, Qmax, platelets, APTT, PT, TT, and fibrinogen after operation, as well as WBC and neutrophils before discharge, were not significantly different from baseline values (p > 0.05).

In addition, we compared mean change in each outcome indicator between groups A and B, and adjusted for age, BMI, PV, history of smoking, history of drinking, SBP, DBP and FBG. Adjusted results showed that there was no significant difference in mean changes for IPSS, Qmax, QoL, WBC, neutrophils, platelets, lymphocytes, APTT, PT, TT, or fibrinogen between the two groups (p > 0.05). The average hospitalization time in group A was longer than that in group B (8.20 ± 3.09 days vs. 6.56 ± 3.60 days, p < 0.01).

**Table 3** summarized the results of univariate and multivariate logistic regression analyses. In unadjusted analysis, not using this drug had no substantial association with transfusion risk (OR = 1.827, 95% CI = 0.764–4.369, p = 0.18). After adjustment for age, BMI, PV, history of smoking, history of drinking, SBP, DBP, and FBG, Hemocoagulase Bothrops Atrox adopting was also not associated with transfusion risk either (OR = 1.582, 95% CI = 0.552–4.538, p = 0.39).

**Table 3:** Effect of Hemocoagulase Bothrops Atrox for inject on blood transfusion during operative.

Transfusion	Group A (n = 443)	Group B (n = 252)	Crude OR (95% CI)	*p*	Adjusted OR (95% CI)*	*p*
No	432(97.52%)	241(95.63%)	1.827(0.764,4.369)	0.176	1.582(0.552,4.538)	0.394
Yes	11(2.48%)	11(4.37%)				

OR, odds ratio; CI, confidence interval.

*, Adjustment for age, body mass index, prostate volume, history of smoking, history of drinking, systolic blood pressure, diastolic blood pressure, and fasting blood glucose.

### Subgroup Analyses

**Figure 1:**
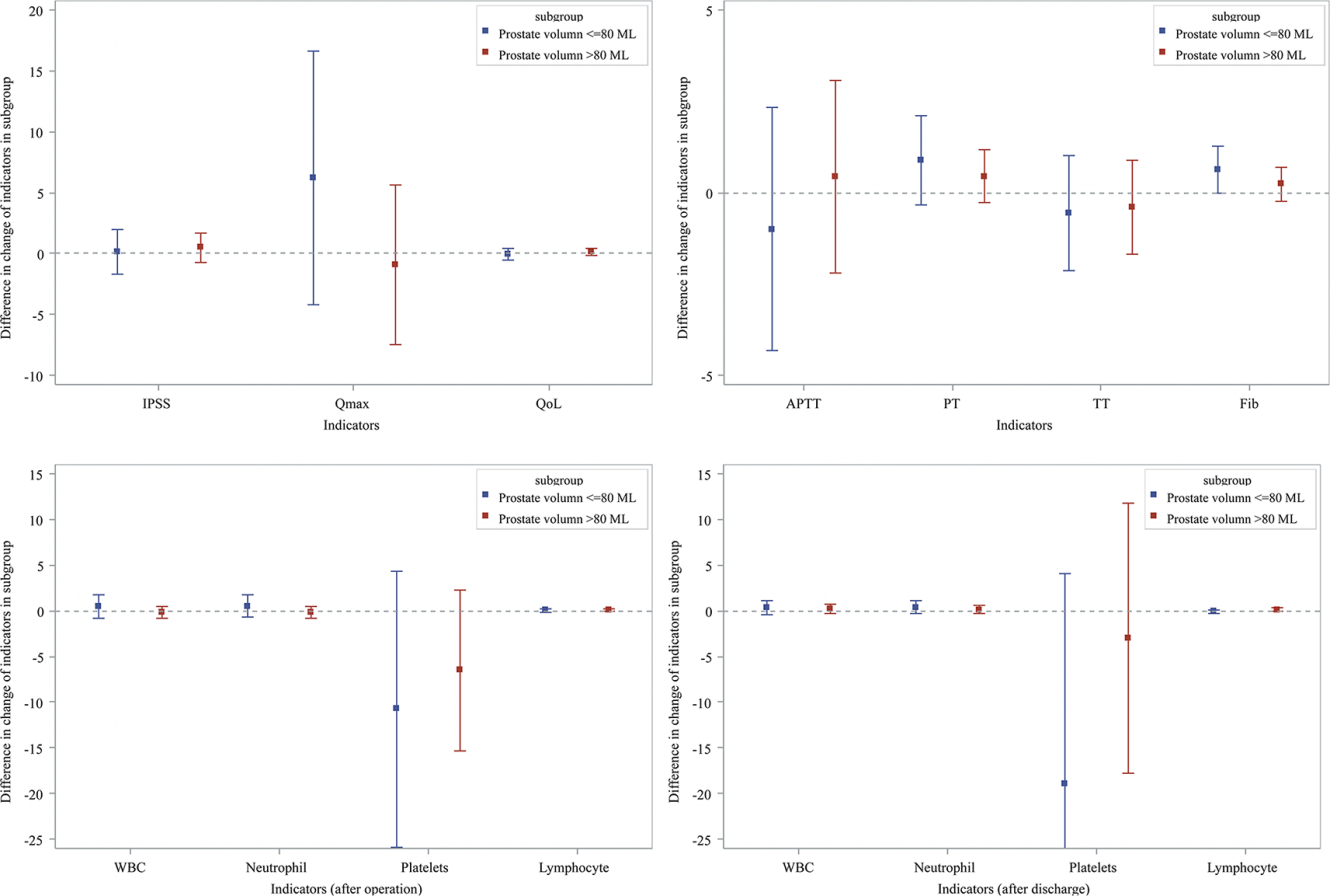
Subgroup analysis effects of Hemocoagulase Bothrops Atrox for inject on prostatic parameters and biochemical indexes.

In order to investigate the influence of PV size, we divided the patients into two groups, PV >80 ml and PV ≤80 ml. **Figure 1** showed prostate indicators, CBC and coagulation screening test results for each subgroup, revealing no significant difference between these two groups.

## Discussion

The study was based on 695 BPH patients receiving TUPKP; of them, 443 did not use hemostatic agents while 252 did during perioperative period. As a result, IPSS, QoL, WBC, neutrophils and lymphocytes in both two groups and PT in group A showed significantly alterations after operation. There was no significant difference between the two groups in Qmax, platelets, APTT, TT, or fibrinogen or in PT for group B after operation. Furthermore, CBC before discharge was compared with the baseline. The results showed that WBC and neutrophils in group A before discharge were higher than baseline figures. There were significant differences in platelets and lymphocytes of the two groups from the baseline, but not in WBC or neutrophils in group B from the baseline.

BPH, a benign urinary system disease, is affected by many factors, and its prevalence increases with age in males (Berry et al., 1984; Park et al., 2018). It is one of the common diseases in middle-aged and elderly men, and causes a huge burden (Saigal and Joyce, 2005; Speakman et al., 2015). An epidemiological survey on BPH in rural areas in Zhengzhou (a city in central China) showed that the prevalence of BPH was 10.04%, and with age increasing this figure increased from 2.17% in individuals aged 40–44 years to 31.11% in those 80years or older (Yue et al., 2019), which were consistent with findings from a meta-analysis (Wang et al., 2015b) and a survey in Europe (Foster et al., 2015). Main clinical symptoms refer to LUTS, including frequent micturition, urinary urgency, urination inability, trouble in urination starting, etc (Martin et al., 2011; Ho et al., 2018). The process of BPH is slow, and early symptoms are not typical. As the lower urinary tract obstruction worsens, symptoms gradually become apparent. Appropriate treatments are generally selected based on patient’s LUTS and decline in QoL, including conservative treatment, pharmacological treatment and surgical treatment (Gravas et al., 2018). Surgery is widely used as an effective way to treat BPH. Surgical methods include open prostatectomy (Saluja et al., 2018), transurethral vaporization of the prostate (TUVP) (Littlejohn et al., 2004), TUPKP (He et al., 2016; Zeng et al., 2018a), and prostate laser surgery (Ketan and Prashant, 2016; Jhanwar et al., 2017).

The TURP may have multiple complications, the most common is bleeding; hence, in many cases, blood transfusion may be needed (Mebust et al., 1989; Doll et al., 1992; Berger et al., 2004). The most terrible complication of conventional TURP is TURS. The incidence of TURS varies greatly from 0.18% to 10.9% (Koshiba et al., 1995). TUPKP is one of the most widely used methods in treating BPH (Martis et al., 2008; Autorino et al., 2009; Muslumanoglu et al., 2012; Li et al., 2015). At present, TUPKP has great advantages in reducing the incidence of traditional TURP complications. Many studies have confirmed the application of plasma bipolar systems in TURP (Botto et al., 2001; Dincel et al., 2004). A number of studies have shown that plasma bipolar systems are as effective as traditional TURP in treating symptoms associated with BPH, and are safer in perioperative and postoperative complications. In a French study, 42 patients with BPH were enrolled. At 3 months after TUPKP, the mean Qmax increased from 7.9 to 19.7 mL/s, and the score for IPSS decreased from 16 to 9. There was no postoperative bleeding event, the average indwelling urethral catheter time was 1.4 days, and the average hospitalization time was 2.2 days (Botto et al., 2001). In addition, some studies have shown that TUPKP can reduce the adverse effects of surgery on patients, such as the catheterization time reduced 44%, hospitalization time decreased 43%, acute complications decreased 52%, and long-term complications reduced 30% (Starkman and Santucci, 2005). However, in a one-year follow-up study, the efficacy and safety of TURP and TUPKP were compared, there was no significant difference in the residual volume, Qmax, IPSS, QoL, hospitalization time and catheterization time between the two groups. The proportion of reinsertion was higher in the TUPKP group (30% vs. 5%). In TURP group, the clot evacuation rate was higher (19% vs. 0), because of the hemostatic advantage of TUPKP (Dunsmuir et al., 2003). The results of several studies showed that the operation time of TUPKP is about 39 minutes to 56 minutes, the intraoperative bleeding volume is 144 mL to 174 mL, and the incidence of postoperative bleeding is 1.47% to 3.3% (El-Shaer et al., 2017; Liu et al., 2017; Zou et al., 2018). According to several meta-analyses, the probability of TUPKP requiring blood transfusion is 1.14% to 1.85% (Mamoulakis et al., 2009; Li et al., 2015; Wang et al., 2015a). Although it has the advantages of less trauma and less bleeding (Chen et al., 2014), bleeding is still a problem that cannot be ignored (Chen et al., 2013). Because the hyperplastic prostate tissue is contained in a dense capsule, it seems to accumulate high pressure in the prostate tissue, which may lead to continuous bleeding after the blood vessels are cut during the prostatectomy (Berger et al., 2005).

The technology of TUPKP is relatively mature. In our research, IPSS and QoL showed significant changes after operation. But for elderly patients, some phenomenon such as poor vascular function may cause prolonged bleeding time, and the chance of developing postoperative complications increases. Hence, during perioperative period, hemostatic agents may be used to prevent or treat bleeding from surgical wounds. Hemostatic agents are widely used in clinical practice, such as abdominal surgery (Wei et al., 2010), orthopedic surgery (Qiu et al., 2017), oral surgery (Joshi et al., 2014), etc. In addition, hemostatic agents are often used to prevent or treat perioperative bleeding in prostatectomy. A study in the United States analyzed the using rate of hemostatic agents in patients undergoing major surgery who were recorded in the Perspective database from 2000 to 2010 (Wright et al., 2014). The results showed that the use of hemostatic agents increased year by year in the past 10 years, reaching 35.2% in 2010. The use of hemostatic agents in prostatectomy was 13.7%, while the transfusion rate for prostatectomy decreased to 6.6%. Common hemostatic agents contained Hemocoagulase Agkistrodon (Wei et al., 2010), Hemocoagulase Bothrops Atrox (Petretski et al., 2000), and Hemocoagulase Atrox for Injection (Zhou, 2017). Hemostatic agents’ functioning is a complex process. Hemostatic agents improve hemostasis through stimulating fibrin formation or inhibiting fibrinolysis (Levi et al., 2002).

According to the results of coagulation screening test after operation, we found an interesting phenomenon: PT was prolonged in patients who did not use hemostatic agents, while postoperative PT in patients who used hemostatic agents did not change significantly from baseline. Therefore, Hemocoagulase Bothrops Atrox for Inject may shorten the activation time of prothrombin, accelerate thrombin formation, activate thrombin-activated fibrinolytic inhibitors and promote blood coagulation. An in vitro experiment (Li et al., 2018) was conducted to explore the mechanism of Hemocoagulase, and the results showed that Hemocoagulase could promote blood coagulation via hydrolyzing fibrinogen into easily absorbed regions and inhibit the activation of plasminogen, thereby reducing the risk of thrombosis.

The prostate has an extensive plexus of venous sinuses, so bleeding risk exists during or after surgery. Although massive hemorrhage shows very low incidence rate, it is still a serious complication of TURP (Xie et al., 2012). When massive hemorrhage occurs, transfusion is an effective treatment. Reportedly, the transfusion rate is about 0.9–2.9% (Reich et al., 2008; Ahmad et al., 2016). The absence of hemostatic agents in this study did not increase the chances of blood transfusion. The reasons may be complex. On the one hand, it may be related to the low rate of massive bleeding of BPH. Most patients experienced little intraoperative or postoperative bleeding volume. After physical treatment, such as catheter compression, better therapeutic effect can be obtained (Bah and Green, 2018). On the other hand, TURP and TUPKP are both the standard operation for BPH. Their effectiveness and safety have been verified by a large number of clinical trials and applications, and the incidence of massive hemorrhage caused by surgery is relatively low (Qian et al., 2017; Babayan, 2018; Jiang and Qian, 2019).

In addition, there are some studies that focus on the effectiveness and safety of TUPKP applications to specific populations. Some studies have performed TUPKP on 193 high-risk elderly patients. The intraoperative blood loss is 42.6 ± 19.5mL, the risk of postoperative secondary bleeding is 1.6%, and the risk of postoperative blood transfusion is about 0.5% (Lv et al., 2012). For patients with large gland, there are many studies reported that the postoperative hemoglobin decreased from 0.65 to 1.63g/dL (Huang et al., 2015; Zhang et al., 2015; Wu et al., 2016), and there is still a greater risk of bleeding during the operation. For patients taking anticoagulants, results show that TUPKP can greatly improve LUTS of patients, and is a safe and effective minimal treatment option, but its reported blood transfusion rate is 2.2%, higher than that of ordinary patients (Li et al., 2015; Wang et al., 2015a; El-Shaer et al., 2017). Interestingly, the results of this study show that use of Hemocoagulase Bothrops Atrox can reduce the hospitalization time without altering the patient’s coagulation parameters and the results of the complete blood count. The reliability of the results of this study was further verified: Hemocoagulase Bothrops Atrox is safe for patients with BPH who undergoing TUPKP. Therefore, in clinical practice, clinicians can prevent thrombosis by using Hemocoagulase Bothrops Atrox in a preventive manner according to the patient’s physical condition, such as high-risk elderly patients, patients with large gland, and patients taking anticoagulants. The use of Hemocoagulase Bothrops Atrox can reduce the hospitalization time, decrease the incidence of blood loss and postoperative complications, and is more in line with the concept of enhanced recovery after surgery (ERAS) (Azhar et al., 2016).

In this study, we noticed that the effect of TUPKP has a lot to do with the surgeon’s proficiency. In advanced hospitals, the surgeon is more skilled in the operation of TUPKP and has a good surgical effect. In the primary hospitals, the surgeon’s operational proficiency still needs to be further improved, and TUPKP still needs further promotion. Our study also had some limitations. First of all, the sample size was not large enough, probably not sufficient to produce powerful evidence, which might be false negative or positive. Secondly, this study only included subjects from one region in China, and the results may not be suitable for people in other regions or ethnic groups. This study adopted an observational design that may be affected by many factors, further weaken our findings. Next, no in-depth analysis was performed.

In conclusion, findings from this study suggest that whether to use Hemocoagulase Bothrops Atrox for Inject or not has no influence on the clinical parameters of BPH patients undergoing TUPKP. Moreover, adopting Hemocoagulase Bothrops Atrox for Inject can shorten prothrombin time, hospitalization time and probably might be safe among BPH patients undergoing TUPKP, achieving fine hemostasis and coagulation efficacy, and was not influenced by the size of prostate volume. High quality and larger samples studies should be operated, especially randomized controlled trials, to explore short-term and long-term effects in future.

## Data Availability Statement

The analyzed data sets generated during the study are available from the corresponding author on reasonable request.

## Ethics Statement

The studies involving human participants were reviewed and approved by Committee for Ethical Affairs of the Zhongnan Hospital of Wuhan University. The patients/participants provided their written informed consent to participate in this study.

## Author Contributions

B-HL, X-HW, and HZh designed this study. Z-JY, C-YW, X-DL and X-YR collected data. T-ZL re-checked data. B-HL and HZi performed analysis. B-HL and Z-JY wrote the manuscript. X-HW and HZh reviewed the manuscript.

## Funding

This work was supported by the National Key Research and Development Plan of China (2016YFC0106300) and Technical Innovation Major Program of Hubei province (2016ACA152), without any financial interest or benefit.

## Conflict of Interest

The authors declare that the research was conducted in the absence of any commercial or financial relationships that could be construed as a potential conflict of interest.
